# Mapping of afferent and efferent connections of phenylethanolamine *N*‐methyltransferase‐expressing neurons in the nucleus tractus solitarii

**DOI:** 10.1111/cns.14808

**Published:** 2024-06-17

**Authors:** Mengchu Zhu, Shirui Jun, Xiaojun Nie, Jinting Chen, Yinchao Hao, Hongxiao Yu, Xiang Zhang, Lu Sun, Yuelin Liu, Xiangshan Yuan, Fang Yuan, Sheng Wang

**Affiliations:** ^1^ Department of Neurobiology Hebei Medical University Shijiazhuang Hebei China; ^2^ Department of Laboratory Diagnostics Hebei Medical University Shijiazhuang Hebei China; ^3^ Department of Anatomy and Histoembryology, School of Basic Medical Sciences Fudan University Shanghai China; ^4^ Department of Neurology Jinshan Hospital Affiliated to Fudan University Shanghai China; ^5^ Hebei Key Laboratory of Neurophysiology Shijiazhuang Hebei Province China

**Keywords:** mapping, neural circuit, nucleus tractus solitarii, PNMT

## Abstract

**Objective:**

Phenylethanolamine *N*‐methyltransferase (PNMT)‐expressing neurons in the nucleus tractus solitarii (NTS) contribute to the regulation of autonomic functions. However, the neural circuits linking these neurons to other brain regions remain unclear. This study aims to investigate the connectivity mechanisms of the PNMT‐expressing neurons in the NTS (NTS^PNMT^ neurons).

**Methods:**

The methodologies employed in this study included a modified rabies virus‐based retrograde neural tracing technique, conventional viral anterograde tracing, and immunohistochemical staining procedures.

**Results:**

A total of 43 upstream nuclei projecting to NTS^PNMT^ neurons were identified, spanning several key brain regions including the medulla oblongata, pons, midbrain, cerebellum, diencephalon, and telencephalon. Notably, dense projections to the NTS^PNMT^ neurons were observed from the central amygdaloid nucleus, paraventricular nucleus of the hypothalamus, area postrema, and the gigantocellular reticular nucleus. In contrast, the ventrolateral medulla, lateral parabrachial nucleus, and lateral hypothalamic area were identified as the primary destinations for axon terminals originating from NTS^PNMT^ neurons. Additionally, reciprocal projections were evident among 21 nuclei, primarily situated within the medulla oblongata.

**Conclusion:**

Our research findings demonstrate that NTS^PNMT^ neurons form extensive connections with numerous nuclei, emphasizing their essential role in the homeostatic regulation of vital autonomic functions.

## Graphical abstract

This schematic diagram illustrating the whole brain inputs and outputs of NTS^PNMT^ neurons.

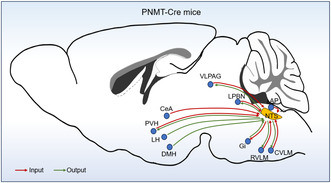

## 1 INTRODUCTION

The nucleus tractus solitarii (NTS) receives visceral sensory information and integrates these information to orchestrate autonomic functions such as blood pressure,^1^ breathing^2^ and gut motility.^3^ The NTS not only integrates information from peripheral receptors, but also receives inputs from central nervous systems, such as brainstem,^4^ hypothalamus,^5^ and limbic system.^6^ Meanwhile, NTS neurons extend their axons to numerous brain regions,^7^, ^8^, ^9^ endowing the NTS with the remarkable ability to modulate multiple physiological functions. In addition to regulating cardiopulmonary activity,^2^, ^9^ compelling evidence has also linked NTS neurons to the regulation of sleep,^10^ feeding^11^ and anorexia.^12^ It is noteworthy that these complex functions are orchestrated via circuit‐ and neuronal phenotype‐specific mechanisms, affirming the multifunctional role of the NTS in homeostasis and the variety in its neuronal functionality.

NTS neurons consist of glutamatergic, GABAergic, catecholaminergic and glycinergic neurons. Among them, catecholaminergic NTS neurons can be further classified into two subtypes based on their expression patterns of different biomarkers. Noradrenergic neurons, labeled with tyrosine hydroxylase (TH) and/or dopamine beta‐hydroxylase (DβH), are called A2 neurons, while adrenergic neurons expressing phenylethanolamine *N*‐methyltransferase (PNMT) are termed C2 neurons.^13^ Catecholaminergic neurons in the NTS have been implicated in autonomic, endocrine, cognitive, and behavioral functions.^14^, ^15^, ^16^ Recent studies focused on the regulation of hypoglycemic hunger by TH‐expressing noradrenergic neurons,^11^ and blood glucose levels and anorexia by DβH‐expressing noradrenergic neurons.^12^ PNMT‐expressing neurons in the NTS (hereafter called NTS^PNMT^ neurons) has proven to participate in regulating the hypoglycemic response.^17^ Furthermore, the neurotransmitters released from these neurons, such as glutamate, norepinephrine, and adrenaline, have a diverse range of downstream effects on animal behaviors, including anxiety, stress, reward, feeding, and cardiovascular function.^18^, ^19^, ^20^, ^21^, ^22^ Our latest findings have identified neuronal phenotype‐ and circuit‐specific mechanisms underlying control of blood pressure by NTS^PNMT^ neurons.^23^ Despite their significant roles in multiple physiological processes, the anatomical and functional connections of NTS^PNMT^ neurons remain largely unknown.

Based on this lack of knowledge, in the present study, both a modified rabies virus tracing vector and a Cre‐dependent adeno‐associated virus (AAV) anterograde tracing vector were utilized in a PNMT‐Cre mouse line to selectively target NTS^PNMT^ neurons. We sought to comprehensively map both afferent and efferent connections of NTS^PNMT^ neurons throughout the brain and provide a structural framework to facilitate the understanding of the neural mechanisms underlying the regulation of blood pressure, breathing and metabolism.

## 2 MATERIALS AND METHODS

### 2.1 Animals

All experiments were performed in accordance with the Guide for the Care and Use of Laboratory Animals, and were approved by Animal Care and Ethical Committee of Hebei Medical University (#Hebmu‐2019001). Mice of either sex, aged at least 8 weeks, were selected for the experiments. The PNMT‐Cre mice were kindly presented by Professor Ming Lei (University of Oxford), as validated before.^24^ R26R‐EYFP mice (stock No. 006148) were purchased from the Jackson Laboratory (Bar Harbor, ME, USA). Mice were kept under a 12 h:12 h light: dark cycle with ad libitum access to food and water.

### 2.2 Preparation of viral vectors

For the rabies virus tracing, AAV‐EF1α‐DIO‐TVA‐GFP, AAV‐EF1α‐DIO‐RVG and RV‐EnvA‐ΔG‐dsRed were packaged by Brain Case (Wuhan, China). Prior to injections, AAV‐EF1α‐DIO‐TVA‐GFP (1.7 × 10^13^ genome copies/mL) and AAV‐EF1α‐DIO‐RVG (6.8 × 10^13^ genome copies/mL) were mixed at a ratio of 1:2. The titer of RV‐EnvA‐ΔG‐dsRed was approximately 5.0 × 10^13^ fluorescence‐forming units/mL. For anterograde tracing experiments, rAAV‐EF1α‐DIO‐EYFP‐WPRE‐PA (2.5 × 10^12^ genome copies/mL) (cat no. PT‐0012; Brain Case, Wuhan, China) was used to label PNMT neurons.

### 2.3 Stereotaxic surgery

Viral vectors and surgical procedures used in this study were described as previous study.^9^ Briefly, mice were anesthetized with pentobarbital sodium (5% pentobarbital sodium, 1.5 mL/kg) and placed in a stereotaxic apparatus (RWD, Life Science, Shenzhen, China). A small hole was drilled in the skull while carefully monitoring for corneal and hindpaw withdrawal reflexes. The body temperature was maintained at 37°C with a programmed‐controlled heating pad, and the eyes were covered with ophthalmic ointment to prevent drying. An aseptically prepared virus‐filled glass micropipette (~25 μm diameter) connected to a pressure‐driving syringe pump (Harvard Instruments, Holliston, MA, USA) was placed within the NTS using the following coordinates: anterior/posterior 0.2 mm, medial/lateral ±0.3 mm, dorsal/ventral −0.2 mm; anterior/posterior 0.3 mm, medial/lateral ±0.4 mm, dorsal/ventral −0.3 mm. For rabies virus tracing, AAV‐EF1α‐DIO‐TVA‐GFP and AAV‐EF1α‐DIO‐RVG (80 nL) were initially injected, followed by injections of of RV‐EnvA‐ΔG‐dsRed (100 nL) 2 weeks post‐injection. For the injection of modified rabies virus, a second procedure was performed as described above, injecting 100 nL of RV‐EnVA‐ΔG‐dsRed. Histological sections were prepared 7–10 days after the second surgery. For anterograde tracing experiments, rAAV‐EF1α‐DIO‐EYFP‐WPRE‐PA (80 nL) was injected into the NTS using a syringe pump, and mice were given a 4‐week recovery period prior to subsequent immunohistochemical experiments.

### 2.4 Single‐cell RT‐PCR

PNMT‐Cre‐EYFP mice were anesthetized 1–2 weeks after receiving only helper AAV injections, and transcardial perfusion was performed using ice‐cold modified artificial cerebrospinal fluid (aCSF) saturated with 95% O_2_ and 5% CO_2_, which contained the following components (in mM): 260 sucrose, 26 NaHCO_3_, 10 glucose, 5 MgCl_2_, 3 KCl, 1.25 NaH_2_PO_4_, 1 KA, and 1 CaCl_2_. The brains were then rapidly removed, and acute coronal slices (300 μm) containing the NTS were cut on a vibratome (VT1200; Leica, Wetzlar, Germany) in ice‐cold modified aCSF. Next, slices were transferred to a holding chamber containing normal recording aCSF (in mM): 125 NaCl, 26 NaHCO_3_, 25 glucose, 2.5 KCl, 2 CaCl_2_, 1.25 NaH_2_PO_4_, and 1.0 MgSO_4_, and allowed to recover for 30 min. Enzymatic resolution was performed by incubating the slices in trypsin solution (0.5 mg/mL) at room temperature for 60 min, followed by being placed in a cell culture plate and washed with PIPE solution (in mM): 25 D‐glucose, 1 CaCl_2_·H_2_O, 5 KCl, 20 PIPE, 120 NaCl, and 1 MgCl·6H_2_O and incubated at room temperature for 60 min. Then, slices were maintained at room temperature for 30 min before recording. Slices were submerged in a recording chamber and superfused with aCSF (2 mL/min) at 30–32°C. Slices were visualized using a fixed‐stage upright microscope (BX51W1, Olympus, Japan) equipped with a 40× water immersion objective and an infrared‐sensitive CCD camera. The NTS^PNMT^ neurons were identified based on their EYFP expression, and the cytosolic contents were aspirated into a patch pipette and expelled into a 200 μL PCR tube as previously described.^23^ The single‐cell reverse‐transcription PCR (RT‐PCR) protocol was designed to detect the presence of mRNA coding for PNMT. Reverse transcription and PCR amplification were performed with gene‐specific multiplex primer using the SuperScript III One‐Step RT‐PCR kit (catalog number: 12574018, ThermoFisher). The reaction was performed as follows: 30 min at 55°C, 2 min at 94°C; 70 cycles of 20 s at 94°C, 30 s at 61°C, and 25 s at 68°C; and 5 min at 68°C. The PCR products were visualized by electrophoresis in agarose gels (1.5%) with ethidium bromide. The expected size of each final PCR product was PNMT 139 bp. The specific primers for PNMT gene, which were custom‐designed and synthesized (Biosune Biotechnology, Shanghai), were as follows: PNMT‐F primer, 5′ to 3′: CAGACCTGAAGCACGCTACAG; PNMT‐R primer, 5′ to 3′: TAGTTGTTGCGGAGATAGGCG.

### 2.5 Fluorescent in situ hybridization

We performed fluorescent in situ hybridization (FISH) using RNAScope Multiplex Reagent Kits (ACDBio, USA) on fresh frozen sections. Frozen sections (10 μm thickness) were cut and air‐dried inside the cryostat for less than 20 min. Following this, sections were fixed in ice‐cold 4% formaldehyde for 15 min and subsequently dehydrated through a graded ethanol series. The slices were treated with H_2_O_2_ for 10 min and washed in PBS. Protease III was added and incubated at room temperature for 20 min. The RNA probes utilized in this experiment targeted TH and DβH. Slices were incubated with the RNA probes in the HybEZ humidified incubator for 2.5 h at 40°C, washed with ACD buffer and sequentially incubated in reagents AMP1‐FL and AMP2‐FL for 30 min, followed by AMP3‐FL for 15 min. Diamidino‐2‐phenylindole (DAPI) was used for subsequent staining. Confocal images were acquired with a confocal microscope (LSM 800, Carl Zeiss, Jena, Germany).

### 2.6 Histology

Mice were injected with viruses as described above. One week after injection of the modified rabies virus or 4 weeks after injection of AAV‐EF1α‐DIO‐EYFP, mice were subjected to deep anesthesia using urethane (1.8 g/kg, intraperitoneally) and subsequently perfused transcardially with saline, followed by 100 mL of 4% formaldehyde in 0.1 M phosphate buffer (PB, pH 7.4). The brains were removed, post‐fixed for 24 h at 4% PFA at 4°C, then cryoprotected in 30% PBS‐buffered sucrose solution until the brainstem was saturated (24–36 h). Tissues were embedded in OCT compound, and stored at −80°C before use. The brains were coronally sectioned at a thickness of 25 μm on a cryostat (CM1950; Leica Microsystems, Wetszlar, Germany) in three series and were collected in 0.01 M phosphate‐buffered saline (PBS, pH 7.4). Brain sections were blocked in 5% bovine serum albumin (BSA) in PBS (0.25% Triton X‐100 in PBS) for 30 min at room temperature (23–24°C), followed by incubation with primary antibodies in 2% BSA–PBS overnight at 4°C. Subsequently, the sections were washed with PBS (3 × 5 min) and incubated with fluorescent secondary antibodies at room temperature for 2 h, followed by incubation with DAPI (1:3000, cat. #D9542; Sigma‐Aldrich, St. Louis, MO, USA) at room temperature for 10 min. All rinses and incubations were done over a shaker at low speed. After rinsing with PBS (3 × 5 min), sections were mounted on slides with Vectashield Antifade Mounting Medium (Vector Laboratories, Burlingame, CA, USA) for visualization.

### 2.7 Image analysis

Images were acquired using a laser‐scanning confocal microscope (LSM 800). Cell enumeration was carried out manually based on the confocal images. FISH was used to determine neurochemical phenotypes. Analysis of both input neurons and axonal terminals, as well as the size of input or output regions, was conducted utilizing ZEN software using ZEN software (Carl Zeiss, Jena, Germany) and quantified using ImageJ (U.S. National Institutes of Health, Bethesda, MD, USA). The proportion of input neurons within each nucleus was determined by calculating the ratio of the normalized count of dsRed‐labeled cells in each nucleus to the overall count of dsRed‐labeled cells. According to the proportion of cells in each nucleus, we defined three grades of afferent inputs as numerous input (>5%), moderate input (2%–5%), and minimal input (<2%). To quantitative analysis of outputs of NTS^PNMT^ neurons, axonal varicosities were counted using ImageJ. The ratio of the average number of varicosities to the total count of whole‐brain projection varicosities was computed to assess the fraction of axonal projections. Axons with transverse diameters exceeding 0.5 mm were classified as varicose. Classification of all axonal varicosities was divided into three categories: dense (over 5%), moderate (2%–5%), and sparse (<2%). Statistical analysis was performed using Prism8 (RRID:SCR_002798; GraphPad Software, San Diego, CA, USA), and graphical representations were created with Adobe Illustrator 2020 (Adobe Systems, San Jose, CA, USA). All data are expressed as mean ± standard error of the mean (SEM).

The primary antibodies used were as follows: Chicken anti‐GFP (dilution 1:1000, Abcam, catalog# ab13970), Rabbit anti‐TH (dilution 1:1000, EMD millipore, catalog# MAB152), Rabbit anti‐Orexin Receptor 1 (dilution 1:500, Abcam, catalog# ab68718), Sheep anti‐ChAT (dilution 1:200, Abcam, catalog# ab18736), Rabbit anti‐FOXP2 (dilution 1:500, Abcam, catalog# ab16046), Rabbit anti‐pSTAT3 (dilution 1:500, Cell Signaling Technology, catalog #9145s), Rabbit anti‐nNOS (dilution 1:500, Cell Signaling Technology, catalog #4231), Mouse anti‐CaMKIIa (dilution 1:500, Gene Tex, catalog #GTX41976), NK‐1R (dilution 1:300, Abcam, catalog #11219342). Fluorophore‐conjugated secondary antibodies used were: Cy™3 AffiniPure Goat Anti‐Rabbit IgG (H + L) (dilution 1:500, Jackson ImmunoResearch Laboratories, catalog# 111‐165‐003), Goat polyclonal Secondary Antibody to Chicken IgY‐H&L (Alexa Fluor® 488) (dilution 1:500, Abcam, catalog# ab150169), Donkey anti‐Mouse IgG H&L (Alexa Fluor® 555) (dilution 1:500, Abcam, catalog# ab150106).

## 3 RESULTS

### 3.1 Distribution of PNMT‐expressing neurons within the NTS

**FIGURE 1:**
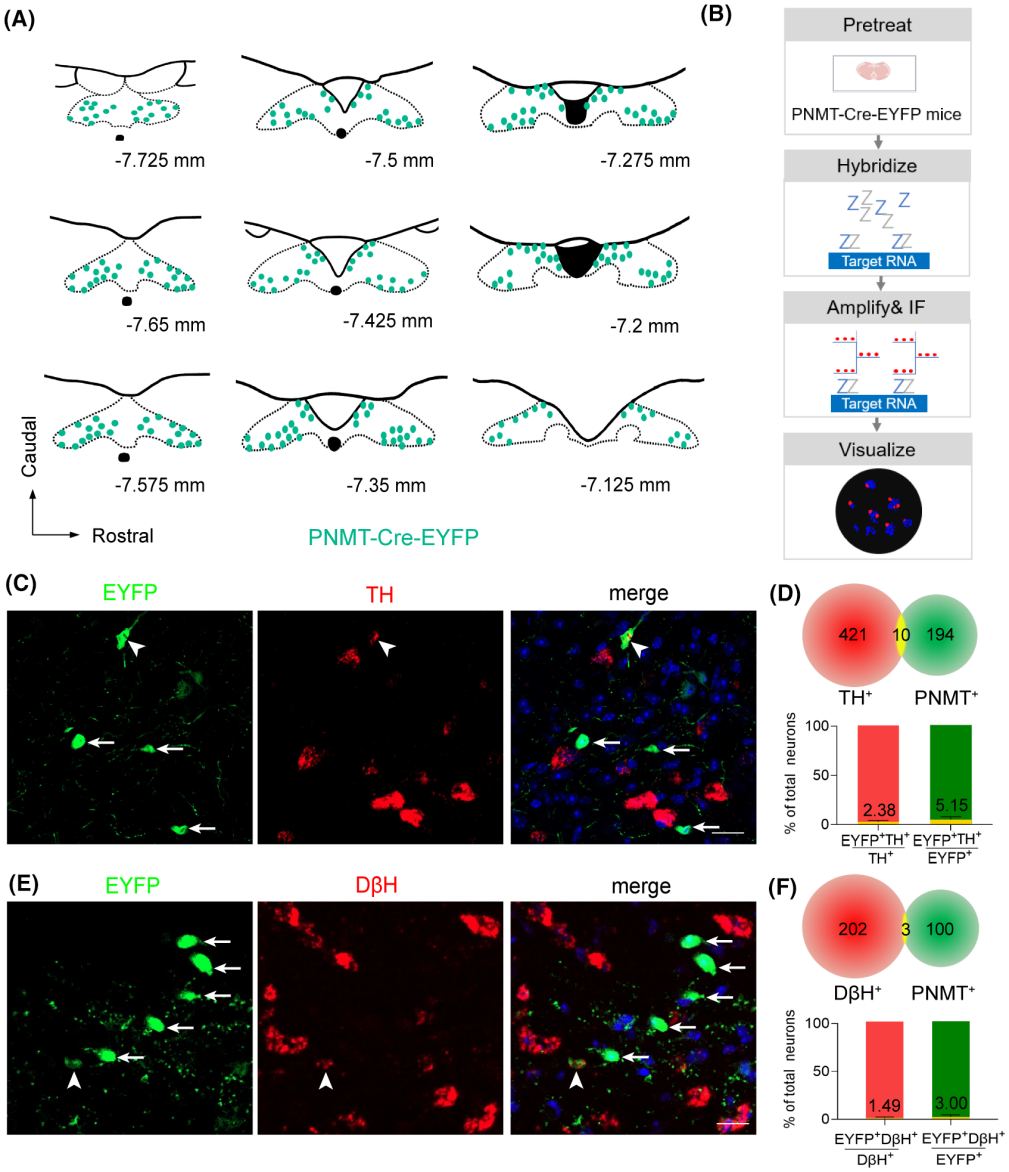
Distribution and chemical phenotypes of NTS^PNMT^ neurons. (A) Schematic of rostrocaudal distribution of PNMT‐expression neurons (EYFP^+^) in the NTS from a PNMT‐Cre‐EYFP transgenic mouse. PNMT‐expression neurons were pinpointed in each coronal plane based on immunohistochemical detection. (B) Schematic of a experimental preceduare for fluorescent in situ hybridization. (C) Photomicrographs showing the co‐expression of TH RNA (red) and PNMT (green) in the NTS. Blue, DAPI. The neuron indicated by the white arrowhead is the PNMT neuron with co‐expression of TH, while the neurons indicated by the white arrow are PNMT neurons without the co‐expression of TH. Scale bar, 20 μm. (D) Quantification of the co‐expression of TH RNA and PNMT (*n* = 3 samples, from 3 mice). (E) Photomicrographs showing the co‐expression of DβH RNA (red) and PNMT (green) in the NTS. Blue, DAPI. The neuron indicated by the white arrowhead is the PNMT neuron with the co‐expression of DβH, while the neurons indicated by the white arrows are PNMT neurons without the co‐expression of DβH. Scale bar, 20 μm. (F) Quantification of the co‐expression of DβH RNA and PNMT (*n* = 3 samples, from 3 mice).

To delineate the spatial distribution of PNMT neurons within the NTS, a transgenic mouse model, PNMT‐Cre::R26R‐EYFP, was generated, specifically targeting PNMT‐expressing neurons through the crossbreeding of PNMT‐Cre mice with Cre‐dependent R26R‐EYFP mice. EYFP expression was subsequently observed in numerous neurons distributed along the caudal to rostral extent of the NTS (Figure 1A). Previous studies have suggested that the largest population of PNMT neurons in the central nervous system resides in the ventrolateral medulla (VLM, called C1 neurons), where TH and DβH, two molecular markers, are also expressed.^25^ The residual contingent of PNMT‐expressing neurons is predominantly dispersed within the NTS. However, uncertainties persist regarding the concurrent expression of TH and DβH by NTS^PNMT^ neurons. To further check the co‐expression pattern of both molecules in NTS^PNMT^ neurons, we used the FISH technique to label these neurons in PNMT‐Cre‐EYFP mice (Figure 1B). Our findings delineated that only a small subset of PNMT neurons within the rostral NTS exhibited co‐localization with either TH or DβH immunoreactive signals, with a prevalence of 5.15% and 3.00%, respectively (Figure 1C–F). These neurochemical phenotypic differences suggest that NTS^PNMT^ neurons may have potential variations in neural circuitry and functional characteristics compared to A2 or C1 neurons.

### 3.2 Visualization of local monosynaptic inputs to NTS^PNMT^ neurons

**FIGURE 2:**
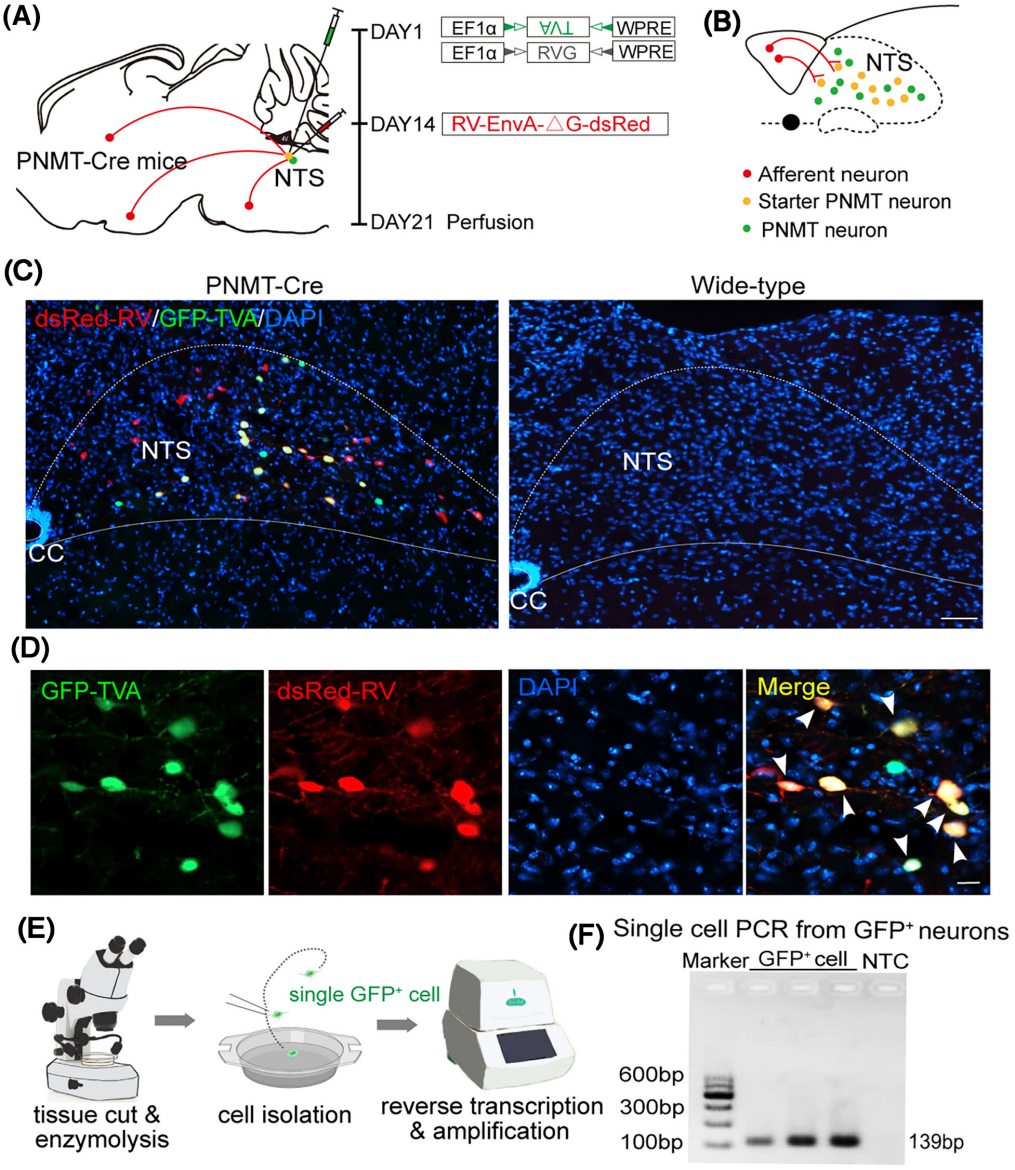
Validation of PNMT‐expressing neurons and input neurons within the NTS. (A) Schematic of viral strategy for transsynaptic retrograde labeling of NTS^PNMT^ neurons in a PNMT‐Cre mouse line. The rabies virus was genetically modified by pseudotyping with EnvA (RV‐EnvA‐ΔG‐dsRed) and was administered 14 days after injections of the helper viruses including AAV‐EF1α‐DIO‐TVA‐GFP and AAV‐EF1α‐DIO‐RVG. (B) Illustration of fluorescent neurons: GFP‐expressing NTS^PNMT^ neurons, dsRed‐expressing input neurons and starter neurons with the co‐expression of GFP and dsRed. (C) Immunohistochemical detection of GFP‐expressing PNMT neurons, input neurons and starter neurons in the NTS from a PNMT‐Cre mouse (left) but not in a wild‐type mouse (right). Scale bar, 100 μm. (D) Typical magnified images showing GFP‐expressing PNMT neurons (green), input neurons (red) and starter neurons (yellow, white arrowhead). Scale bar, 20 μm. (E) Diagram showing single cell RT‐PCR analysis of PNMT expression in GFP‐expressing neuron from NTS of PNMT‐Cre mouse. (F) Gel images of single cell RT‐PCR reaction confirming PNMT expression in GFP‐labeled neurons of the NTS. GAPDH was taken as negative control (right).

To elucidate brain‐wide afferents to NTS^PNMT^ neurons, a specialized experimental approach employing a modified rabies virus in conjunction with the helper AAV was executed targeting NTS^PNMT^ neurons derived from a PNMT‐Cre mouse line. The injection protocol involved the administration of helper viruses, specifically AAV‐ EF1α‐DIO‐TVA‐GFP and AAV‐EF1α‐DIO‐RVG, into the unilateral NTS (*n* = 4 mice). Following 2 weeks to facilitate viral expression, the RV‐EnvA‐ΔG‐dsRed was injected into the same region, selectively infecting TVA‐expressing cells and enabling the retrograde transport into presynaptic neurons (Figure 2A). Based on this tracing strategy, we were able to identify GFP‐expressing NTS ^PNMT^ neurons (green), dsRed‐expressing input neurons (red) and starter neurons with the co‐expression of GFP and dsRed (Figure 2B–F). Notably, when this injection protocol was replicated in wild‐type mice, which lacked Cre recombinase expression, neither GFP nor dsRed fluorescence was observed, confirming the specificity of the Cre‐dependent system (Figure 2C, right panel). Additionally, single‐cell RT‐PCR analysis revealed that expression of PNMT RNA was detected in GFP‐positive neurons in the NTS (80%, *n* = 16 positive cells/20 total cells. Figure 2E,F), further substantiating the authenticity and functionality of the PNMT‐Cre mouse line in targeting the relevant neuronal population.

### 3.3 Monosynaptic inputs onto NTS^PNMT^ neurons in the whole brain

**FIGURE 3:**
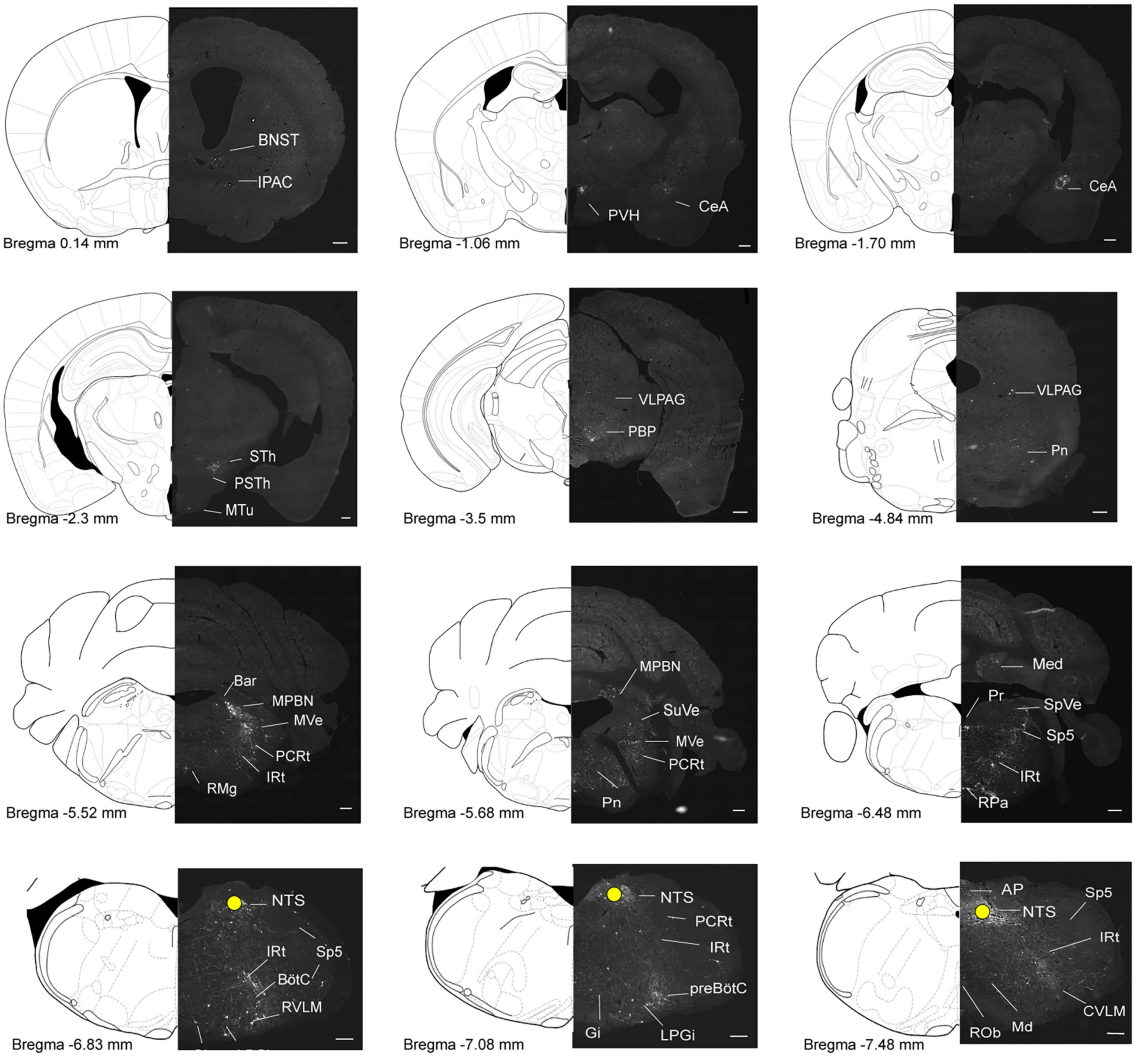
Representative images of monosynaptic inputs to NTS^PNMT^ neurons from the whole brain. Regions are labeled according to the Mouse Brain Atlas. Scale bars, 200 μm. AP, area postrema; Bar, Barrington's nucleus; BNST, bed nucleus of the stria terminalis; BötC, Bötzinger complex; CeA, central amygdaloid nucleus; CVLM, caudal ventrolateral medulla; Gi, gigantocellular reticular nucleus; IPAC, interstitial nucleus of the posterior limb of the anterior commissure; IRt, intermediate reticular nucleus; LPGi, lateral paragigantocellular nucleus; Md, medullary reticular nucleus; Med, medial (fastigial) cerebellar nucleus; MPBN, medial parabrachial nucleus; MTu, medial tuberal nucleus; MVe, medial vestibular nucleus; NTS, nucleus tractus solitarii; PBP, parabrachial pigmented nucleus; PCRt, parvocellular reticular nucleus; Pn, pontine reticular nucleus; Pr, prepositus nucleus; preBötC, pre‐Bötzinger complex; PSTh, parasubthalamic nucleus; PVH, paraventricular hypothalamus; RMg, raphe magnus nucleus; ROb, raphe obscurus nucleus; RPa, raphe pallidus nucleus; RTN, retrotrapezoid nucleus; RVLM, rostral ventrolateral medulla; Sp5, spinal trigeminal nucleus; SpVe, spinal vestibular nucleus; STh, subthalamic nucleus; SuVe, superior vestibular nucleus; VLPAG, ventrolateral periaqueductal gray.

**FIGURE 4:**
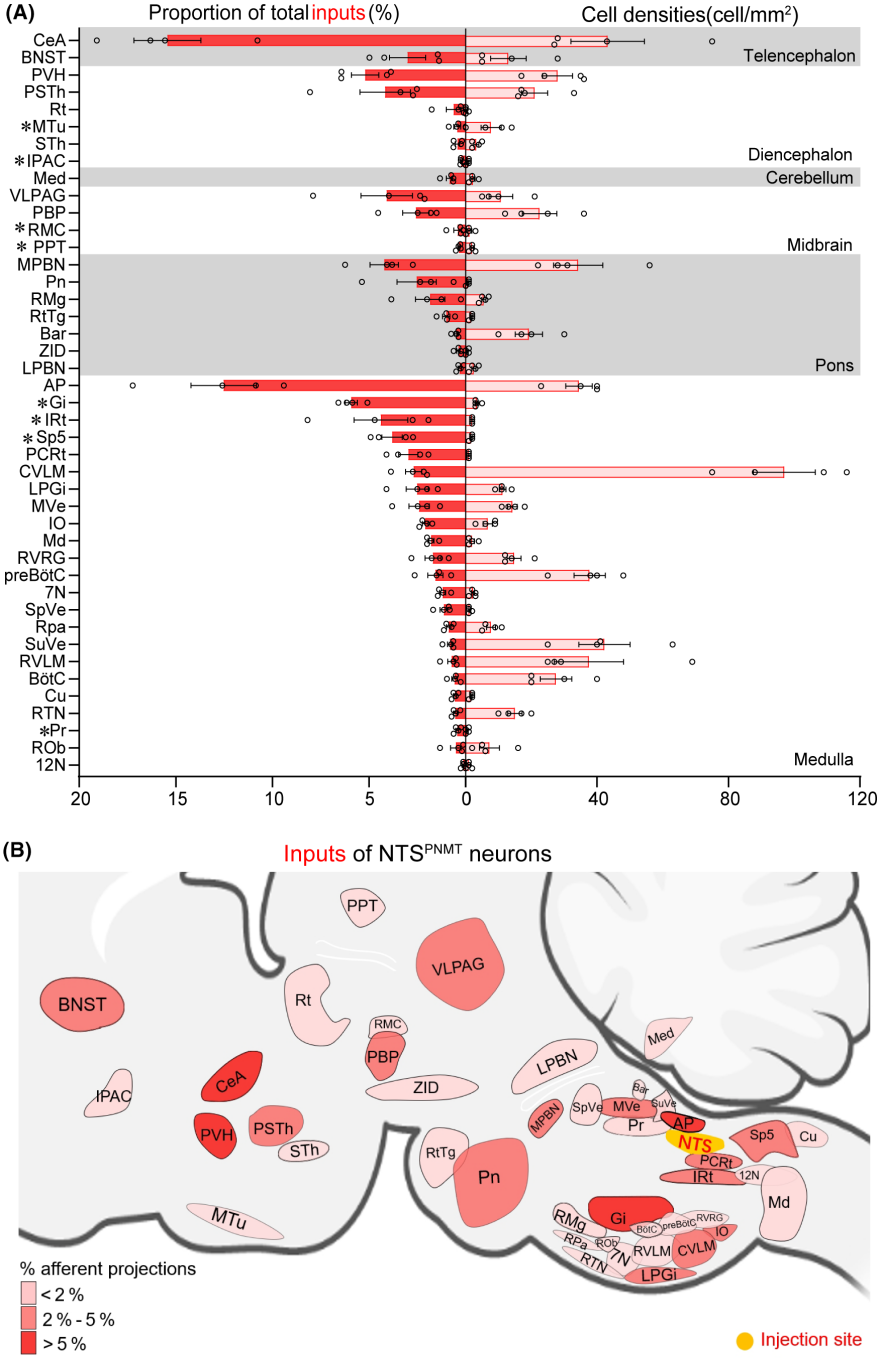
Summary of monosynaptic inputs to NTS^PNMT^ neurons. (A) Quantitative analysis of whole‐brain afferent nuclei of NTS^PNMT^ neurons. Error bar represents the SEM (*n* = 4). Circles represent individual animal values. Brain areas are grouped into six general structures: the telencephalon, diencephalon, cerebellum, midbrain, pons, and medulla oblongata. Asterisks represent the unilateral inputs. The proportion along with the cell density of input neurons to the NTS^PNMT^ neurons in each brain area is shown. (B) Summary of the afferent projection profiles of NTS^PNMT^ neurons. 7N, facial nucleus; 12N, hypoglossal nucleus; AP, area postrema; Bar, Barrington's nucleus; BNST, bed nucleus of the stria terminalis; BötC, Bötzinger complex; CeA, central amygdaloid nucleus; Cu, cuneate nucleus; CVLM, caudal ventrolateral medulla; Gi, gigantocellular reticular nucleus; IO, inferior olive; IPAC, interstitial nucleus of the posterior limb of the anterior commissure; IRt, intermediate reticular nucleus; LPBN, lateral parabrachial nucleus; LPGi, lateral paragigantocellular nucleus; Md, medullary reticular nucleus; Med, medial (fastigial) cerebellar nucleus; MPBN, medial parabrachial nucleus; MTu, medial tuberal nucleus; MVe, medial vestibular nucleus; PBP, parabrachial pigmented nucleus; PCRt, parvocellular reticular nucleus; RMg, raph e magnus nucleus; Pn, pontine reticular nucleu; PPT, posterior pretectal nucleus; Pr, prepositus nucleus; preBötC, pre‐Bötzinger complex; PSTh parasubthalamic nucleus; PVH, paraventricular hypothalamus; RMC, red nucleus, magnocellular part; ROb, raphe obscurus nucleus; RPa, raphe pallidus nucleus; Rt, reticular thalamic nucleus; RTN, retrotrapezoid nucleus; RtTg, reticulotegmental nucleus of the pons; RVLM, rostral ventrolateral medulla; RVRG, rostral Ventral Respiratory Group; Sp5, spinal trigeminal nucleus; SpVe, spinal vestibular nucleus; STh, subthalamic nucleus; SuVe, superior vestibular nucleus; VLPAG, ventrolateral periaqueductal gray; ZID, zona incerta, dorsal part.

**FIGURE 5:**
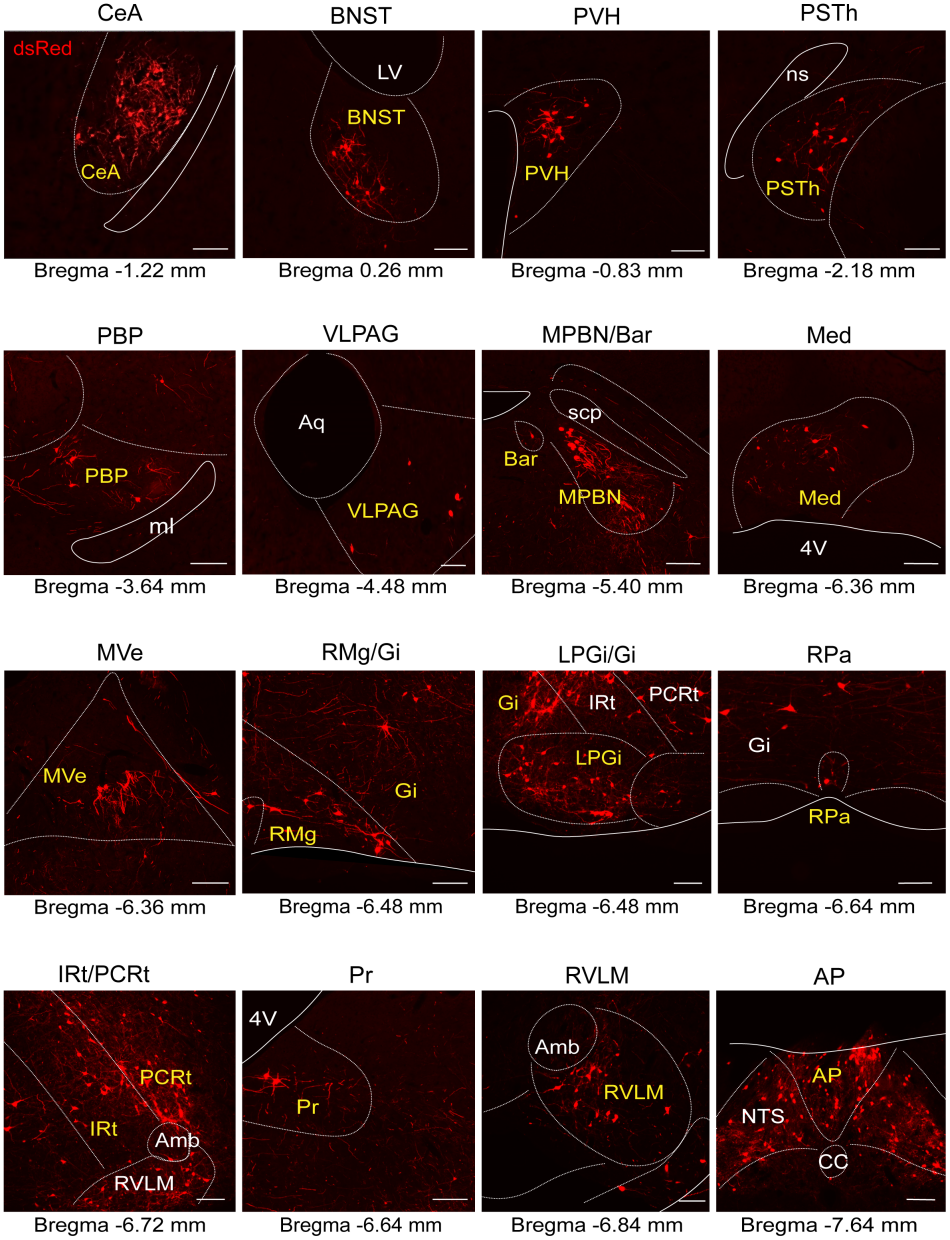
Representative images of selected brain regions with monosynaptic inputs to NTS^PNMT^ neurons. Scale bars, 100 μm. 4V, 4th ventricle; Amb, ambiguus nucleus; AP, area postrema; Aq, aqueduct (Sylvius); Bar, Barrington's nucleus; BNST, bed nucleus of the stria terminalis; CC, central canal; CeA, central amygdaloid nucleus; Gi, gigantocellular reticular nucleus; IRt, intermediate reticular nucleus; LPGi, lateral paragigantocellular nucleus; LV, lateral ventricles; Med, medial (fastigial) cerebellar nucleus; ml, medial lemniscus; MPBN, medial parabrachial nucleus; MVe, medial vestibular nucleus; ns, nigrostriatal bundle; NTS, nucleus tractus solitarii; PBP, parabrachial pigmented nucleus; PCRt, parvocellular reticular nucleus; Pr, prepositus nucleus; PSTh parasubthalamic nucleus; PVH, paraventricular hypothalamus; RMg, raphe magnus nucleus; RPa, raphe pallidus nucleus; RVLM, rostral ventrolateral medulla; scp, superior cerebellar peduncle; VLPAG, ventrolateral periaqueductal gray.

To explore the whole‐brain distribution of monosynaptic afferents to NTS^PNMT^ neurons, we observed serial brain coronal sections in four PNMT‐Cre mice, respectively. Coronal slices were integrated with an anatomical atlas to show the detailed distribution of presynaptic neurons relative to NTS^PNMT^ neurons throughout the brain (Figure 3). In total, there were 43 upstream nuclei innervating NTS^PNMT^ neurons. DsRed‐positive neurons were mainly located throughout six brain regions, including the medulla, pons, midbrain, cerebellum, diencephalon, and telencephalon (Figure 4). To evaluate the distribution of input nuclei, we counted the cell numbers and cell density at different brain nuclei (Figure 4A). The cell numbers were calculated by the ratio of the number of dsRed‐positive neurons in each nucleus to total dsRed‐labeled neurons in the whole brain. To calculate cell density, we divided the number of dsRed‐positive cells in each nucleus by the corresponding nucleus area. The dsRed‐positive afferent neurons were mostly found in nuclei of the medulla oblongata when compared with other five regions. Surprisingly, a large number of neurons within the telencephalon also innervated NTS^PNMT^ neurons. We found that PNMT neurons received dense afferents (>5% of total input neurons) from 4 nuclei: the central amygdaloid nucleus (CeA, 15.43%), paraventricular hypothalamus (PVH, 5.20%), area postrema (12.51%) and Gi (5.91%). Among them, the CeA had the strongest projection to NTS^PNMT^ neurons. In addition, we have further identified that CeA neurons innervate NTS^PNMT^ neurons using anterograde tracing viral strategies (Figure S1). Moreover, we found 13 nuclei with moderate inputs (2%–5% of total input neurons) such as the bed nucleus of stria terminalis (BNST) in the telencephalon, parasubthalamic nucleus in the diencephalon, periaqueductal gray (PAG) in the midbrain, medial parabrachial nucleus in the pons, intermediate reticular nucleus (IRt), spinal trigeminal nucleus and parvocellular reticular nucleus (PCRt) in the medulla oblongata. We also found a few dsRed‐positive neurons (<2% of total input neurons) in other 26 areas as shown in Figure 4A. NTS^PNMT^ neurons mainly receive afferent projections from both hemispheres. The clusters of nuclei receiving unilateral inputs are marked with asterisks (Figure 4A). Given the individual differences between mice, we did not always detect dsRed^+^ neurons in every same region for different rabies‐injected mouse. To get a handle on this, we summarized all nuclei that have retrogradely labeled neurons. The inputs of NTS^PNMT^ are depicted schematically in Figure 4B, mirroring the upstream nuclei. In order to demonstrate the distribution of dsRed‐labeled presynaptic neurons throughout the entire brain, we presented representative and enlarged images of the inputs (Figure 5).

### 3.4 Outputs of NTS^PNMT^ neurons in the whole brain

**FIGURE 6:**
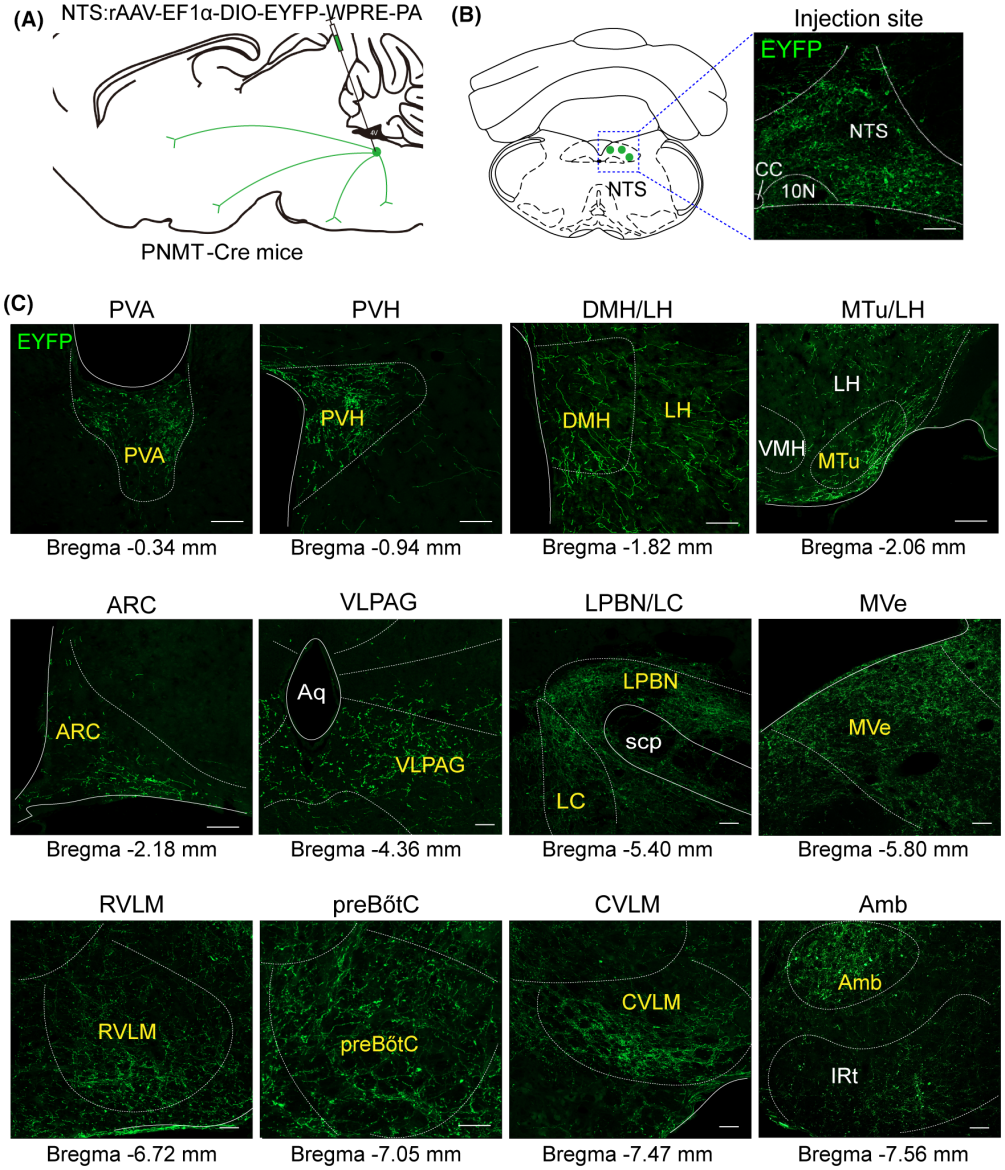
Projections of NTS^PNMT^ neurons. (A) Schematic of anterograde tracing strategy of by injection of a recombinant anterograde vector to the NTS of PNMT‐Cre mice. (B) Typical images showing the expression of fluorescent PNMT neurons (green) in the NTS. Scale Bar, 100 μm. (C) Example distribution of axon terminals of NTS^PNMT^ neurons. Scale bars, 100 μm. Amb, ambiguus nucleus; Aq, aqueduct (Sylvius); ARC, arcuate nucleus; CVLM, caudal ventrolateral medulla; DMH, dorsomedial hypothalamus; IRt, intermediate reticular nucleus; LC, locus coeruleus; LH, lateral hypothalamic area; LPBN, lateral parabrachial nucleus; MTu, medial tuberal nucleus; MVe, medial vestibular nucleus; preBötC, pre‐Bötzinger complex; PVA, paraventricular thalamic nucleus, anterior part; PVH, paraventricular hypothalamus; RVLM, rostral ventrolateral medulla; scp, superior cerebellar peduncle; VLPAG, ventrolateral periaqueductal gray; VMH, ventromedial hypothalamic nucleus.

To study the axonal terminal distribution of NTS^PNMT^ neurons, we injected rAAV‐EF1α‐DIO‐EYFP‐WPRE‐PA into the unilateral NTS in PNMT‐Cre mice (*n* = 8 mice, Figure 6A). Following a 4‐week interval to allow for sufficient viral expression, EYFP was observed delineating both the somata and processes of the NTS^PNMT^ neurons (Figure 6B). In a control experiment, the identical viral vector was introduced into the unilateral NTS of wild‐type mice, which did not express Cre recombinase. In this cohort, no EYFP expression was detected within any neurons (data not shown), underscoring the specificity of the AAV tracing vector for Cre‐expressing cells. Images were captured and compared with the reference brain atlas to reveal the distribution of anterograde projection of NTS^PNMT^ neurons.

**FIGURE 7:**
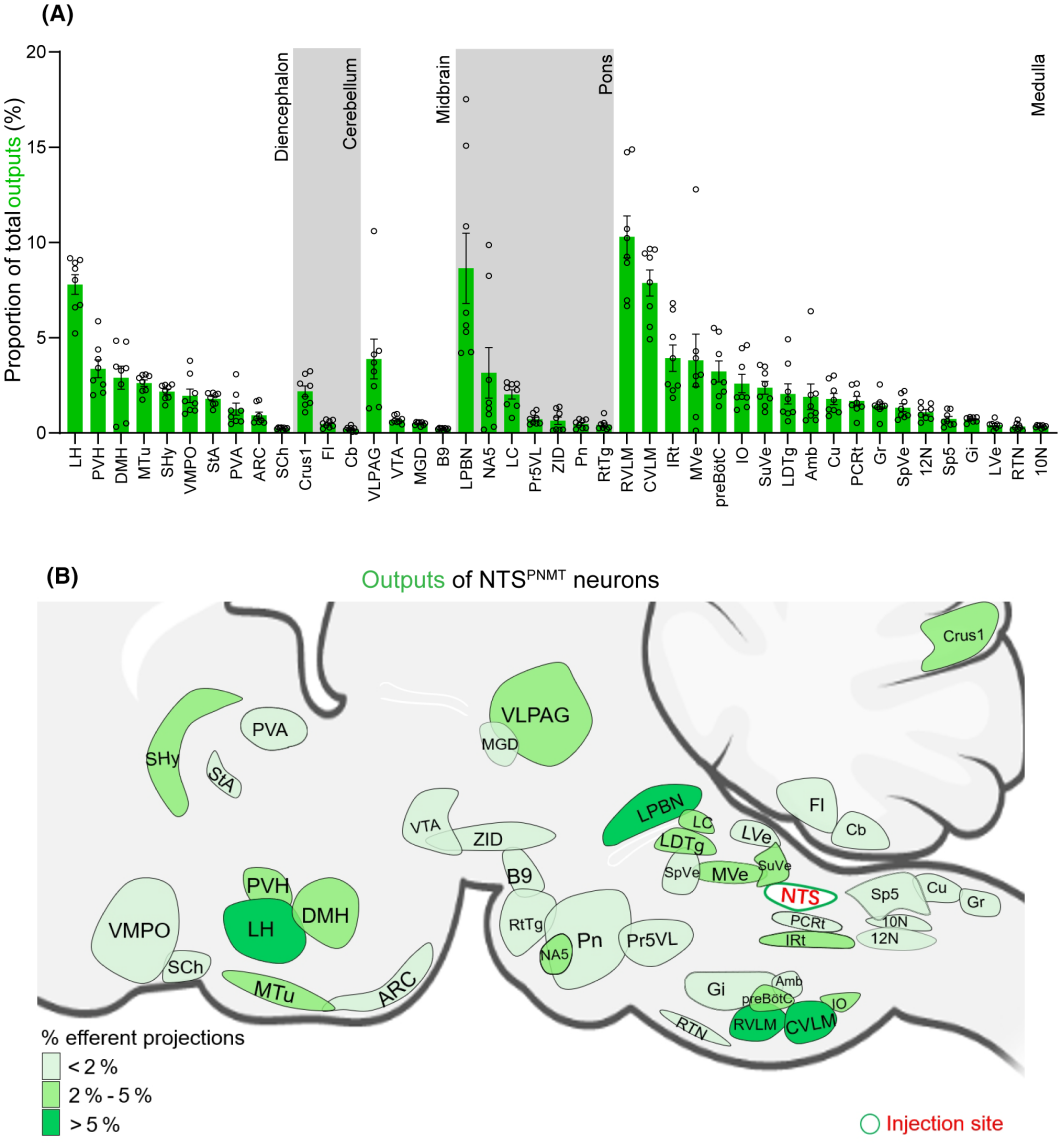
Summary of outputs from NTS^PNMT^ neurons. (A) Statistical analysis of proportion of outputs distributed in each brain region to the total number of neurons in the whole brain. Brain areas are grouped into five general structures: the diencephalon, cerebellum, midbrain, pons, and medulla oblongata. Each error bar represents the SEM. Circles represent individual animal values (*n* = 8). (B) Summary of the major efferent outputs to NTS^PNMT^ neurons. The depth of color represents the location and number of projections of NTS^PNMT^ neurons. 10N, dorsal motor nucleus of vagus; 12N, hypoglossal nucleus; Amb, ambiguus nucleus; ARC, arcuate nucleus; B9, serotonin cells; Cb, cerebellum; Crus1, crus 1 of the ansiform lobule; Cu, cuneate nucleus; CVLM, caudal ventrolateral medulla; DMH, dorsomedial hypothalamus; Fl, flocculus; Gi, gigantocellular reticular nucleus; Gr, gracile nucleus; IO, inferior olive; IRt, intermediate reticular nucleus; LC, locus coeruleus; LDTg, laterodorsal tegmental nucleus; LH, lateral hypothalamic area; LPBN, lateral parabrachial nucleus; LVe, lateral vestibular nucleus; MGD, medial geniculate nucleus, dorsal part; MTu, medial tuberal nucleus; MVe, medial vestibular nucleus; NA5, noradrenalin cells; PCRt, parvocellular reticular nucleus; Pn, pontine reticular nucleus; Pr5VL, principal sensory trigeminal nucleus, ventrolateral part; preBötC, pre‐Bötzinger complex; PVA, paraventricular thalamic nucleus, anterior part; PVH, paraventricular hypothalamus; RTN, retrotrapezoid nucleus; RtTg, reticulotegmental nucleus of the pons; RVLM, rostral ventrolateral medulla; SCh, suprachiasmatic nucleus; SHy, septohypothalamic nucleus; Sp5, spinal trigeminal nucleus, caudal part; SpVe, spinal vestibular nucleus; StA, strial part of the preoptic area; SuVe, superior vestibular nucleus; VLPAG, ventrolateral periaqueductal gray; VMPO, ventromedial preoptic nucleus; VTA, ventral tegmental area; ZID, zona incerta, dorsal part.

Axonal terminals from labeled PNMT neurons were detected across 43 distinct brain regions encompassed within five major areas: the medulla oblongata, pons, midbrain, cerebellum, and diencephalon (Figure 6C). To accurately quantify the efferent projections from PNMT neurons to each respective region, we adopted a systematic counting process focused on the EYFP‐labeled axonal varicosities, ranging from regions of dense to sparse labeling (Figure 7A). It is important to note that in our methodology, axonal shafts devoid of puncta and varicosities were not included in the analysis to avoid miscounting axonal fibers that merely pass through without forming synaptic connections, thereby ensuring a more precise determination of the innervation patterns by PNMT neurons. Our findings reveal that NTS^PNMT^ neurons project bilaterally to downstream nuclei, with a preferential distribution of axons occurring on the ipsilateral side relative to the site of injection. Among the five divisions, the medulla oblongata received the highest proportion (47.93%) of projections from NTS^PNMT^ neurons (Figure 7A). Most of the projections from these neurons were observed in the VLM (18.17%) and the lateral parabrachial nucleus (LPBN, 8.65%). The lateral hypothalamic area (LH, 7.80%) in the diencephalon also housed numerous axon terminals from NTS^PNMT^ neurons. Furthermore, among the various regions, 14 nuclei exhibited a moderate level of axonal terminals, each accounting for 2%–5% of the total terminations, such as the PVH, dorsomedial hypothalamus (DMH), medial tuberal nucleus and septohypothalamic nucleus in the diencephalon, the ventrolateral periaqueductal gray in the midbrain, locus coeruleus (LC) in the pons, along with the IRt, medial vestibular nucleus and preBötC in the medulla oblongata. Additionally, a smaller dispersal of axonal terminals (less than 2% of total output terminals) was noted in 25 other nuclei, as detailed in Figure 7A. To facilitate a comprehensive understanding of the efferential dissemination of NTS^PNMT^ neurons, schematic representations illustratively summarizing the connections to downstream nuclei were prepared and are accessible in Figure 7B.

**FIGURE 8:**
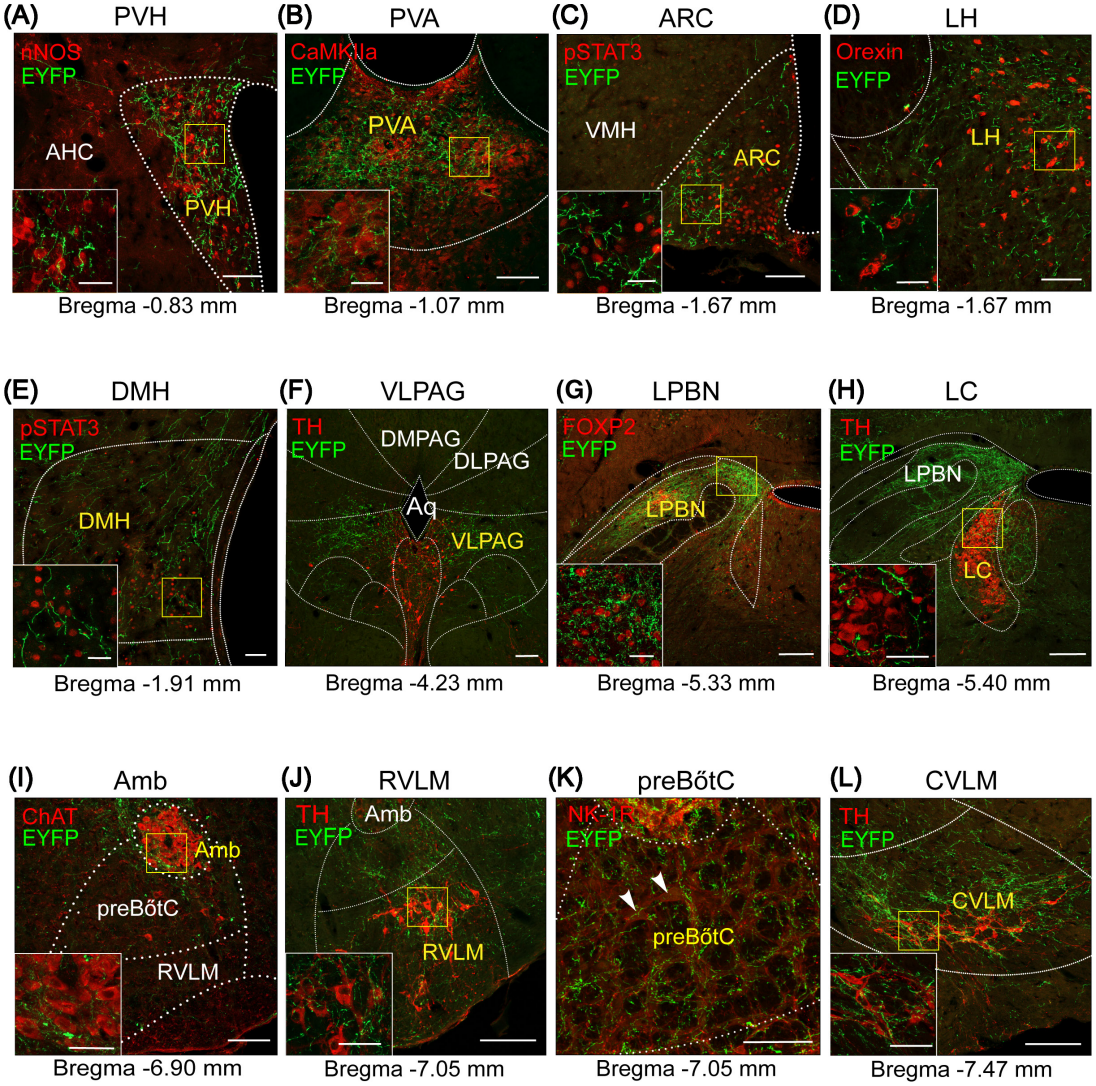
Typical images showing selected nuclei receiving outputs from NTS^PNMT^ neurons. Confocal images showing regional distribution of axonal terminals (green) originating from NTS^PNMT^ neurons. Each brain region was marked by nNOS (red) in the PVH (A), CaMKIIa (red) in the PVA (B), pSTAT3 (red) in the ARC (C) and DMH (E), Orexin (red) in the LH (D), TH (red) in the PAG (F), LC (H), RVLM (J), and CVLM (L), ChAT (red) in the Amb (I), FOXP2 (red) in LPBN (G), and NK‐1R (red) in preBötC (K). Scale bars, 100 μm. The white solid boxes show the magnified fluorescence image of yellow solid boxes of each image. Scale bars, 50 μm. AHC, anterior hypothalamic area, central part; Amb, ambiguus nucleus; Aq, aqueduct (Sylvius); ARC, arcuate nucleus; CVLM, caudal ventrolateral medulla; DLPAG, dorsolateral periaqueductal gray; DMH, dorsomedial hypothalamus; DMPAG, dorsomedial periaqueductal gray; LC, locus coeruleus; LH, lateral hypothalamic area; LPBN, lateral parabrachial nucleus; PAG, periaqueductal gray; preBötC, pre‐Bötzinger complex; PVA, paraventricular thalamic nucleus, anterior part; PVH, paraventricular hypothalamus; RVLM, rostral ventrolateral medulla; VLPAG, ventrolateral periaqueductal gray; VMH, ventromedial hypothalamic nucleus.

In this study, a variety of molecular markers were employed to demarcate the contours of each targeted downstream nucleus (Figure 8). Within the medulla oblongata, the rostral and caudal ventrolateral medulla (RVLM and CVLM), distinguished by their expression of TH, received the most substantial projections from NTS^PNMT^ neurons, comprising 10.30% and 7.87% of the projections, respectively (Figures 7A, 8J,L). The dense axonal terminals of NTS^PNMT^ neurons were also found in the medial vestibular nucleus, which is generally considered to be a key nucleus involved in the regulation of orthostatic blood pressure.^26^ Moreover, moderate projections were directed towards both the ambiguous nucleus, characterized by choline acetyltransferase (ChAT) expression, and the preBötzinger complex (preBötC), marked by neurokinin‐1 receptor (NK‐1R) staining (Figures 7A, 8I,K). In the pons, NTS^PNMT^ neurons sent dense axonal projections to the LPBN, labeled by FOXP2 (Figure 8G), which regulates inspiratory by reducing the inspiratory and expiratory time.^27^ We also found that NTS^PNMT^ neurons have direct projections to noradrenalin cells in the LC, labeled by TH (Figure 8H), which has been shown to be involved in the development of the respiratory system in neonatal mice.^28^ In the midbrain, moderate projections of NTS^PNMT^ neurons to the PAG has been found, labeled by TH (Figure 8F), which is involved in regulating behavioral or mood‐related respiratory activity.^29^ All the above findings suggest the involvement of NTS^PNMT^ neurons in the regulating of cardiorespiratory activity. Additionally, in the thalamus and hypothalamus, the potential roles of NTS^PNMT^ neurons in energy metabolism, circadian rhythm, food intake and thermoregulation are further suggested by the projections to the LH (labeled by Orexin), PVH (labeled by nNOS), dorsomedial hypothalamus (labeled by pSTAT3), arcuate nucleus (ARC, labeled by pSTAT3), and paraventricular thalamic nucleus, anterior part (PVA, labeled by CaMKIIa) (Figure 8A–E). This comprehensive delineation of the whole‐brain projections from NTS^PNMT^ neurons provides a foundational context for further investigations into the physiological roles performed by these neurons in the integration of cardiorespiratory functions with metabolic and behavioral processes.

### 3.5 Analysis of reciprocal neural connectivity of NTS^PNMT^ neurons in the whole brain

**FIGURE 9:**
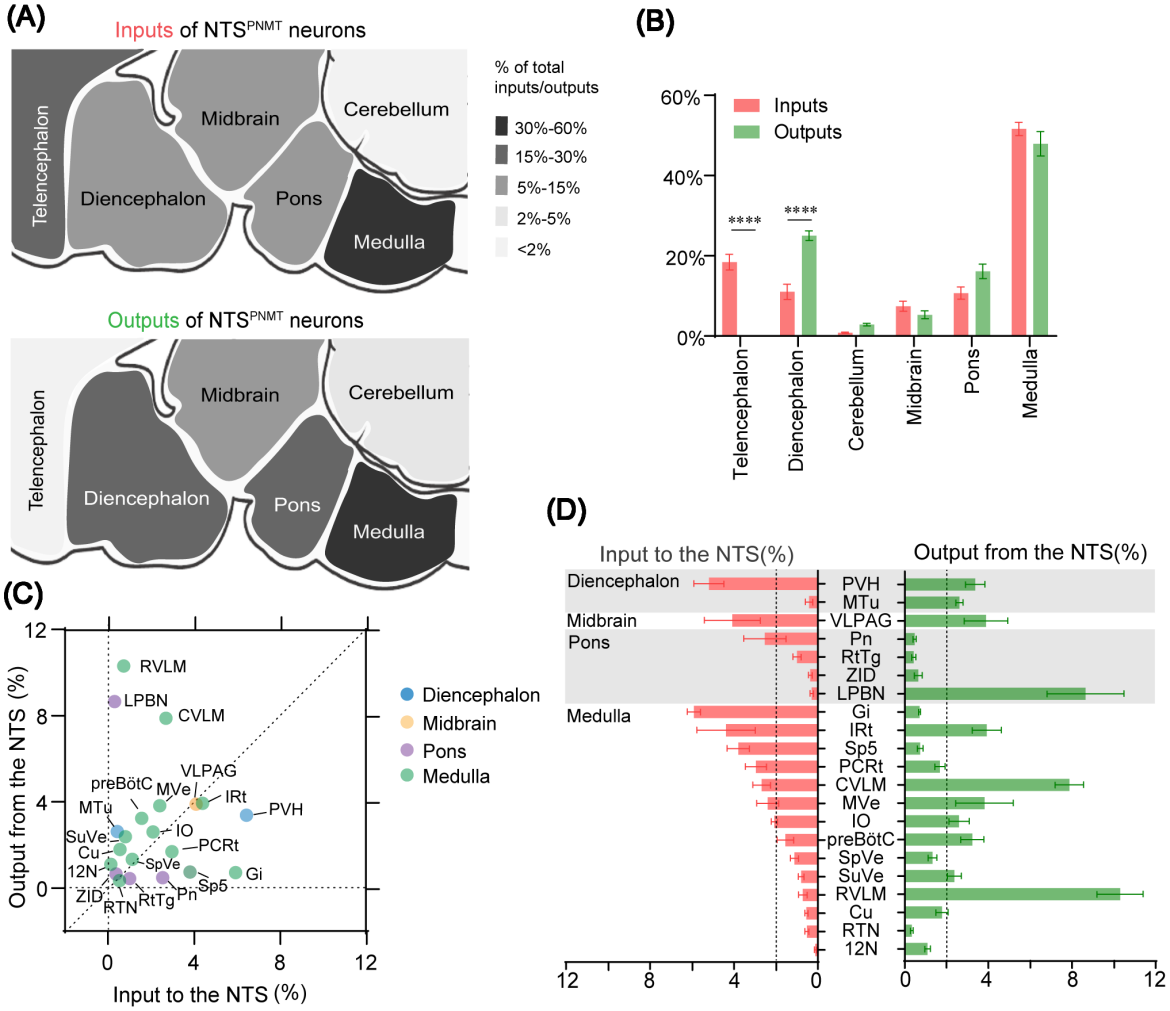
Comparison between inputs and outputs of NTS^PNMT^ neurons. (A) Whole‐brain and output distributions of NTS^PNMT^ neurons compared in six major structural general subdivision. Up: proportion of inputs to NTS^PNMT^ neurons. Down: proportion of outputs from NTS^PNMT^ neurons. (B) Percentage of total inputs to and outputs from the whole‐brain to NTS^PNMT^ neurons. The data represent the mean ± SEM, with *n* = 4 for inputs and *n* = 8 for outputs. Levene's test followed by independent samples Student's ttests and Mann–Whitney U test (input vs. output: Telencephalon, *p* = 0.00001; Diencephalon, *p* = 0.00001; Cerebellum, *p* = 0.9859; Midbrain, *p* = 0.9544; pons, *p* = 0.1923; Medulla, *p* = 0.6140, ****p < 0.0001) were used to indicate statistical differences between inputs and outputs of NTS^PNMT^ neurons in the same subdivision. (C) and (D) The percentage of inputs versus outputs in each region of typical nuclei that connected with the NTS^PNMT^ neurons. Brain areas are grouped into four general structures: the diencephalon, midbrain, pons, and medulla. Error bar represents the SEM, with *n* = 4 for inputs and *n* = 8 for outputs. 12N, hypoglossal nucleus; Cu, cuneate nucleus; CVLM, caudal ventrolateral medulla; Gi, gigantocellular reticular nucleus; IO, inferior olive; IRt, intermediate reticular nucleus; LPBN, lateral parabrachial nucleus; MTu, medial tuberal nucleus; MVe, medial vestibular nucleus; PCRt, parvocellular reticular nucleus; Pn, pontine reticular nucleus; preBötC, pre‐Bötzinger complex; PVH, paraventricular hypothalamus; RTN, retrotrapezoid nucleus; RtTg, reticulotegmental nucleus of the pons; RVLM, rostral ventrolateral medulla; Sp5, spinal trigeminal nucleus, caudal part; SpVe, spinal vestibular nucleus; SuVe, superior vestibular nucleus; VLPAG, ventrolateral periaqueductal gray; ZID, zona incerta, dorsal part.

According to distribution features of both input neurons and axon terminals of NTS^PNMT^ neurons, we assessed these nuclei that establish reciprocal neural connectivity with these neurons. Our results demonstrate that NTS^PNMT^ neurons not only receive substantial inputs from the medulla but also send axonal projections abundantly to the same regions (Figure 9A,B). Additionally, while NTS^PNMT^ neurons receive dense afferent projections from the telencephalon, they do not establish reciprocal projections to this region. Conversely, the diencephalon receives dense projections from NTS^PNMT^ neurons, while exhibiting less inputs to the NTS. (Figure 9A,B). For the individual nucleus, the NTS^PNMT^ neurons establish interactive connections with 21 distinct nuclei, among which 15 nuclei experience moderate to dense levels of input and/or output (>2% of total input neurons/output terminals, Figure 9C,D). The medulla oblongata stands out with the highest number of nuclei, approximately 14, exhibiting reciprocal connections with NTS^PNMT^ neurons. Additional nuclei with reciprocal projections include 4 in the pons, 1 in the midbrain, and 2 in the diencephalon. While NTS^PNMT^ neurons receive inputs from some nuclei, such as the Gi, spinal trigeminal nucleus, PCRt and pontine reticular nucleus, their axonal projections back to these areas are comparatively sparse. On the contrary, the medial tuberal nucleus, LPBN, preBötC, superior vestibular nucleus, and RVLM receive abundant inputs from the NTS but send fewer outputs back to it. Moreover, the PVH, VLPAG, IRt, CVLM, medial vestibular nucleus, and inferior olive not only extensively send efferent projections but also receive considerable afferent innervation from NTS^PNMT^ neurons. Finally, the reticulotegmental nucleus of the pons, dorsal part of zona incerta, spinal vestibular nucleus, cuneate nucleus, retrotrapezoid nucleus, and hypoglossal nucleus only had few reciprocal neural connections to NTS^PNMT^ neurons. Overall, the comprehensive mapping and characterization of these reciprocal neural connections, detailed in Figure 9C,D, furnish valuable insights into the neural circuitry involving NTS^PNMT^ neurons and underscore their potential roles in various physiological processes.

## 4 DISCUSSION

The current study delineates the synaptic connectomics of NTS^PNMT^ neurons by employing cell‐type‐specific anterograde and retrograde tracing methodologies in a PNMT‐Cre mouse model, in line with previously documented protocols.^30^ The data illustrate that NTS^PNMT^ neurons predominantly receive synaptic inputs from the medulla oblongata, especially from the area postrema. In contrast, a relatively minor proportion of inputs originates from other brain regions such as the telencephalon, diencephalon, cerebellum, midbrain, and pons. Furthermore, the medulla oblongata also represents the principal region for axonal outputs of NTS^PNMT^ neurons, with notable projections to the RVLM and CVLM. Moreover, NTS^PNMT^ neurons establish reciprocal neural connections with a multitude of nuclei. Furthermore, the afferents and efferent of NTS^PNMT^ neurons were mostly asymmetric across major nuclei.

### 4.1 Comparison with previous similar studies

Throughout all this time, research on the neural connectivity of the NTS catecholaminergic neurons has always been highly regarded, given its crucial roles in various brain functions. They receive visceral afferents and transmitted information to multiple autonomic units of the medulla, midbrain pontine, thalamus and hypothalamus, and anterior forebrain, such as the CeA, DMH, LH, PVH, ARC, and PAG, to regulate cardiovascular activity,^31^ feeding behavior,^11^ emotional regulation,^32^ and processes such as water intake and salt metabolism.^33^ Unfortunately, there has been no comprehensive neural pathways' analysis of adrenergic neurons expressing PNMT, as one of the important neuronal types within the NTS, which constraining the further in‐depth research into the pivotal role of PNMT neurons in physiological regulation. Current relevant neuroanatomical studies mostly outdated and relied on non‐specific chemical tracers, which were unable to reveal cell‐specific synaptic connections to distinct neurons. For instance, Ricardo and Koh^34^ reported ascending projections from the caudal part of the NTS in rats using anterograde autoradiographic and retrograde horseradish peroxidase (HRP) tracer techniques. They found connections to various brain stem territories, including the PVA and forebrain structures like the BNST and the hypothalamus such as the DMH and ARC. The connections between the PVH and the NTS^PNMT^ neurons in rats were also revealed via a retrograde fluorescent tracer (True Blue) by Sawchenko et al.^35^ These studies have several limitations because the input of specific brain regions to the NTS^PNMT^ neurons is difficult to assess based on whole‐brain mapping. Traditional retrograde tracers cannot identify the inputs to the NTS specific cell types. By precisely targeting NTS^PNMT^ neurons with a modified rabies virus and a recombinant adeno‐associated virus, our work has circumvented the limitations of conventional tracers, and characterized a comprehensive whole‐brain map of NTS^PNMT^ neurons. Furthermore, through performing quantitative analysis of the inputs and outputs, our tracing results not only verified previously reported research results but also provided a thorough understanding of the anatomical‐functional link of NTS^PNMT^ neurons throughout the entire brain.

In addition, previous studies have shown that almost all C1 neurons expressing PNMT exhibit high levels of TH and/or DβH, which NTS^PNMT^ neurons is theoretically thought to contain all the three markers.^36^ However, in this study, we found most NTS^PNMT^ neurons presented no TH expression, which is inconsistent with the hypothesis presented in the previous study.^23^ Moreover, an absence of DβH in majority of NTS^PNMT^ neurons had been detected. These neurons with exclusively PNMT but no TH and/or DβH expression has also been reported in the retina^37^ and some brain areas of rat.^38^ The NTS^PNMT^ neurons may harbor an unconventional pathway for catecholamine biosynthesis, which suggest that NTS^PNMT^ may have different physiological functions compared to PNMT neurons located in other areas. For instance, C1 neurons (the largest cluster of PNMT neurons) were primarily involved in regulating the functions of the cardiovascular and respiratory systems, by controlling blood pressure and heart rate, regulating respiratory rhythm, participating in stress and stress response, modulating the activity of the sympathetic nervous system, and maintaining the balance of the cardiovascular and respiratory systems.^39^ Our recent study indicates that the NTS^PNMT^ neurons are also involved in blood pressure regulation, but unlike the C1 neurons, they are involved in a relatively complex manner: Optogenetic stimulation of NTS^PNMT^ neurons targeting the PVN, LPBN, and CVLM elicits a significant reduction in blood pressure. Conversely, activation of NTS^PNMT^ neurons projecting to the RVLM induces a pronounced increase in blood pressure and a concurrent decrease in heart rate.^23^ These functional differences may relate to the special neurotransmitter expression patterns within the NTS^PNMT^ neurons. The specific mechanism underlying the diminished TH and/or DβH immunoreactivity in NTS^PNMT^ neurons remains unexplored and warrants future investigation for its comprehension. Our neuroanatomical network of these catecholaminergic NTS neurons with separate PNMT expression pattern will, in combination with previously published classic studies, provide new insights on mechanism of catecholamine biosynthesis pathway.

### 4.2 Reciprocal connections between NTS^PNMT^ neurons and cardiorespiratory‐related regions

The NTS plays a crucial role in the autonomic regulation of cardiorespiratory processes. Our previous findings also revealed the role of different NTS subgroups in the circuit‐specific regulation of blood pressure^23^ and breathing.^2^, ^9^ Numerous blood pressure‐regulating regions, including the VLM and PVN, are linked to the NTS, which forms the neuronal circuitry for the feedback regulation of blood pressure. NTS sent direct projections to the VLM, especially the CVLM area to reduce blood pressure, and some projects to the RVLM, but the specific function remains to be clarified.^40^ Our results show that there is a bidirectional projection relationship between the NTS and CVLM, implying that the NTS may be involved in the regulation of blood pressure in the form of this interaction with the CVLM. Moreover, studies have confirmed that during CIH, increased respiratory chemoreceptor activity can increase the sympathetic output of A2 neurons in the NTS to the RVLM, resulting in an increase in blood pressure.^41^ Interestingly, our results exhibit that NTS^PNMT^ neurons have dense projections to the RVLM, which is not ruled out that the PNMT neurons projecting to the RVLM may contribute to regulating blood pressure. Furthermore, another nucleus that participates in the formation of cardiorespiratory regulation loops and tightly connects to the NTS mainly exists in the hypothalamus, particularly in the PVH. Some of projection pathways from the NTS to PVH consist of glutamatergic neurons, which innervate the PVH and surrounding cells, including presympathetic, GABAergic and nNOS‐expressing neurons that mainly involved in the activation of sympathetic nerves.^37^ Activating the catecholaminergic input from the NTS to PVH can promote adaptive changes in cardiopulmonary activity under hypoxia.^42^ In our study, we observed a mass input to NTS^PNMT^ neurons from the PVH and dense projections from NTS to PVH, which further validates the involvement of the NTS^PNMT^ neurons in the regulation of cardiovascular function. Additionally, the CeA is key area in forebrain that involved in the regulation of the cardiovascular reflexes. The CeA receives input from higher centers in the forebrain and has extensive connections with the hypothalamus and the medulla, thus links psychologically induced stress with the autonomic control system. Available evidence suggests that the direct projections from the CeA to barosensitive neurons in the NTS and RVLM constitute a potential network for the control of cardiovascular functions during emotional expression.^43^ Given that numerous projections from the CeA to NTS^PNMT^ neurons have been clarified in our data, it is tempting to speculate that these descending projects from the CeA may modulate activity of NTS^PNMT^ neurons during different states of anxiety, and further influence the baroreceptor reflex control of blood pressure at the level of the NTS, which may warrant further investigation.

In addition to blood pressure, the NTS also modulates breathing. The core component of breathing centers exists in the VLM, and is mainly composed of three adjacent nuclei: the Bötzinger complex, the preBötC and the rostral ventral respiratory group.^44^ Among them, the preBötC complex is the core structure that produces rhythmic breathing, and has inspiratory related neurons. The direct projection of the NTS to the preBötC is one of the main regulatory loops of breathing. The previous research has confirmed that activating the Phox2b‐expressing neurons within the NTS can enhance the central respiratory drive, mainly by activating the NTS‐preBötC neural pathway.^45^ However, we found that although the NTS^PNMT^ neurons have projections to the preBötC, the axons are significantly less than projections to the VLM. In addition, NTS^PNMT^ neurons received few monosynaptic inputs from preBötC, suggesting that these neurons may be less involved in the central regulation of respiration. We also found close interconnexions and branching projections between the NTS and some nuclei in midbrain and pons, such as the LC and PBN. A recent report has verified that the projection loop of Phox2b neurons in the LC to preBötC is directly involved in the regulation of central respiratory activity.^45^ The noradrenergic neurons projecting from the NTS to LC also mediate changes in the plasticity of respiratory movement caused by asphyxia.^46^ Therefore, the large number of axons projecting from NTS^PNMT^ neurons to the LC may also participate in maintaining the stability of central respiratory activity. NTS^PNMT^ neurons have dense projections to the LPBN, and meanwhile received projections from the medial parabrachial nucleus neurons. While NTS^PNMT^ neurons have a role in the regulation of blood pressure through multiple pathways, the present data provide putative circuit mechanisms underlying control of breathing by these neurons.^23^ The aforementioned projection patterns gain new insight into analyzing the control role of NTS^PNMT^ neurons in regulating cardiovascular and respiratory activities.

### 4.3 Emotional/behavioral inputs to NTS^PNMT^ neurons

Emotional stimulation would elicit autonomic responses. Besides receiving peripheral visceral projections from the cardiovascular and respiratory, the NTS establishes multiple central afferent connections to brain regions related to visceral sensory reflex functions.^47^ According to past data, the hypothalamus, insular and entorhinal cortex are the major central regions that project to the NTS.^47^, ^48^ These areas are involved in behavioral modulation.^49^ Recent studies revealed that the NTS‐CeA circuit modulate depression‐like behaviors comorbid to chronic pain.^50^ Moreover, the BNST is emerging as a critical region in multiple psychiatric disorders including anxiety, alcohol and substance use disorders. Increased noradrenergic signaling from the NTS to the BNST is responsible for withdrawal‐induced anxiety during ethanol withdrawal.^51^ Other research has reported that the PVH is related to addiction withdrawal and addiction.^52^ Notably, our results also show that the NTS receives large projections from the CeA, BNST and PVH. The descending inputs from infralimbic neurons to catecholaminergic NTS neurons appear to mediate cortical or hypothalamic modulation of sympathetic and parasympathetic activity and represent a functional link between emotional processing and autonomic outflow. From all above, both previous studies and our findings suggest that NTS^PNMT^ neurons could have a pivotal role in regulating and orchestrating emotional behaviors, which provides an affective‐autonomic basis for further study on possible neural circuits that participate in visceral “mind–body” regulation. Further research in the realms of basic science, clinical studies, and pathological investigations will be crucial in unraveling the exact role and mechanisms through which these neurons operate.

### 4.4 Role of NTS^PNMT^ neurons in the regulation of metabolism

The NTS is an important site for the regulation of metabolism. Previous studies have shown that NTS neurons project to the ARC, where satiety signals are integrated with adiposity signals (namely leptin and insulin) and several hypothalamic and supra‐hypothalamic inputs, thus creating a complex network of neural circuits that elaborate the individual response to a meal.^53^ TH‐expressing NTS neurons targeting the ARC contribute to the regulation of food intake by releasing noradrenergic.^11^ The NTS also receives ghrelin neuron projections from the LH to regulate gastric activity.^54^ On the other hand, activating the Glucagon‐like peptide 1 receptor neural pathway from the NTS to dorsolateral tegmental nucleus can inhibit food intake and regulate energy balance.^55^ Moreover, A2 neurons in the NTS control feeding behaviors via projections to the PVH.^56^ The excessive activation of transient receptor potential vanilloid‐1 channel in the NTS of the animal fed with a high‐fat diet can drive the activation of LPBN neurons, and ultimately act on the median preoptic nucleus to release dynorphins, thereby affecting the activity of brown fat and reducing the body's metabolism.^57^ In our work, obvious projections from NTS^PNMT^ neurons to the LH and ARC were also captured. More than that, some PNMT neurons in NTS projected to the DMH, another vital site related to the regulation of food intake and body metabolism, which further provides evidence that such neurons participate in control of metabolism. Furthermore, our results indicate that NTS^PNMT^ neurons receive a substantial number of afferent projections from the CeA, Gi, PCRt and IRt. These regions have previously been shown to play a role in the regulation of metabolism‐related behaviors, further supporting our findings. For example, the CeA play an important role in regulating the nongenomic effect of aldosterone on rapid sodium intake. Additionally, the Gi, a key hub for the coordination of orienting and locomotor behaviors, is involved in the control of swallowing by projecting to the NTS.^58^ In addition, Phox2b neurons in the PCRt are involved in the motor control of feeding behavior. The abovementioned evidence indicates that the NTS may play a role in the regulation of metabolism‐related behavior, as supported by the present results of both retrograde and anterograde tracing studies.

We have mapped the inputs and outputs of NTS^PNMT^ neurons throughout the brain, and discovered them to be closely interconnected with nuclei located in the medulla oblongata. This suggests the crucial role of NTS^PNMT^ neurons in control of various physiological and pathological functions, including blood pressure, breathing, emotional behavior and metabolism. Also, we have compiled a summary of the physiological behaviors associated with the nuclei that provide input or receive projections from NTS^PNMT^ neurons (Tables 1 and 2). This compilation may assist in enhancing the understanding of the role of NTS in various physiological and behavioral functions.

**TABLE 1:** The proportion (>2%) and involved physiological behaviors of nuclei that input to the NTS^PNMT^ neurons.

Brain region	Nucleus	Proportion (%)	Physiological behaviors	References
Telencephalon	CeA	15.43	Emotion, attention, motivation, memory formation and extinction, anxiety and depression, negative behaviors	43, 50, 59
	BNST	3.00	Defensive, anxiety and addiction, sleep–wake behaviors; reward, social behaviors	51, 60
Diencephalon	PVH	5.20	Hunting; anxiety, stress, respiratory, feeding behaviors	56, 61, 62
	PSTh	4.16	Locomotion; REM sleep; defensive behaviors; fear‐associated hypothermia	^63^
Midbrain	VLPAG	4.08	Pain; offensive/defensive behaviors; respiration antinociceptive; itch‐scratching; emotion; sleep–wake behaviors; HR	64, 65, 66
	PBP	2.57	Trigeminovascular sensory processing	^67^
Pons	MPBN	4.20	Wakefulness; respiration	^68^
	Pn	2.53	Auditory startle reflex	^69^
Medulla	AP	12.51	Nausea‐associated behaviors	^70^
	Gi	5.91	Swallowing reflex; locomotor recovery	58, 71
	IRt	4.38	Postinspiratory activity; swallowing; respiratory‐sympathetic coupling; nose motion	^72^
	Sp5	3.80	Pain; temperature sensations; self grooming	^73^
	PCRt	2.95	Feeding behavior	^74^
	CVLM	2.68	Sleep control; cardiovascular regulation	10, 40
	LPGi	2.50	Locomotion	^75^
	MVe	2.40	Maintaining equilibrium; posture; head position	76, 77
	IO	2.08	Tremor; learning and timing of movements	^78^

**TABLE 2:** The proportion (>2%) and involved physiological behaviors of nuclei that that receive output from NTS^PNMT^ neurons.

Brain region	Nucleus	Proportion (%)	Physiological behaviors	References
Diencephalon	LH	7.80	Sleep–wake behaviors; respiration; appetitive behaviors; offensive/defensive behaviors; pain	79, 80
	PVH	3.37	Hunting; anxiety, stress, respiratory, feeding, behaviors; sleep–wake behaviors; respiration	56, 61, 62
	DMH	2.90	Sleep–wake behaviors; reward, food intake, torpor, thermoregulation	81, 82, 83
	SHy	2.17	Affiliative behavior	^84^
Cerebellum	Crus1	2.19	Social behaviors; sensory hypersensitivity	^85^
Midbrain	VLPAG	3.89	Pain; offensive/defensive behaviors; respiration antinociceptive; itch‐scratching; emotion; sleep–wake behaviors; HR	64, 65, 86
Pons	LPBN	8.65	Pain‐like behavior; feeding; respiration	9, 87, 88
	LC	2.02	Autonomic arousal; memory linking	45, 46, 89
Medulla	RVLM	10.30	Oxidative stress; blood pressure; breathing; sleep	^40^
	CVLM	7.87	Sleep control; cardiovascular regulation	10, 40
	IRt	3.93	Postinspiratory activity; swallowing; respiratory‐sympathetic coupling; nose motion	^72^
	MVe	3.81	Maintaining equilibrium; posture; head position	76, 77
	preBötC	3.23	Breathing	^44^
	IO	2.60	Tremor; learning and timing of movements	^78^
	SuVe	2.37	Meteoropathy	^90^
	LDTg	2.05	Energy balance; reward function; sleep–wake regulation	55, 91

## 5 CONCLUSIONS

Our circuit‐ and cell type‐specific tracing results provide a whole‐brain map of synaptic connectomics of NTS^PNMT^ neurons, in particular quantification of inputs and outputs of these neurons, highlighting reciprocal connections between these neurons with other nuclei. Our findings provide a structural framework for the underlying circuit mechanisms related to certain physiological functions.

## AUTHOR CONTRIBUTIONS

Study design and manuscript drafting/revising: Sheng Wang, Fang Yuan, and Xiangshan Yuan. Running of the experiments, acquisition of the data, and figure drawing: Mengchu Zhu, Shirui Jun, Xiaojun Nie, Jinting Chen, and Yinchao Hao. Acquisition of the data and statistical analysis: Hongxiao Yu, Xiang Zhang, Lu Sun, and Yuelin Liu. All authors have read and approved the final version of the manuscript.

## CONFLICT OF INTEREST STATEMENT

The authors declare that there are no conflicts of interest.

## Supporting information


Figure S1.

## Data Availability

The data that support the findings of this study are available from the corresponding author upon reasonable request.
