# Supplementary material for: Mapping of afferent and efferent connections of phenylethanolamine N‐methyltransferase‐expressing neurons in the nucleus tractus solitarii
Source: CNS Neurosci Ther. 2024 Jun 17;30(6):e14808. doi: 10.1111/cns.14808 (PMC11183208; doi:10.1111/cns.14808)
Supplement: Supplementary file 1 — Figure S1. [file CNS-30-e14808-s001.zip › Figure S1.docx]

Figure S1 CeA neurons innervate NTSPNMT neurons. (A) Schematic of viral strategy for using anterograde tracing virus AAV-hsyn-mCherry to validate the innervation of CeA neurons to NTSPNMT neurons. (B) Axon terminals from CeA neurons were observed in the NTS. Arrowheads indicated the CeA axonal terminals probably innervating NTSPNMT neurons. Scale bar, 20 µm. (C) Schematic of viral strategy for validating the innervation of CeA neurons to NTSPNMT neurons utilizing anterograde transsynaptic tracing virus AAV1. (D) The NTSPNMT neurons that directly innervated by CeA neurons were observed in the NTS. Arrowheads indicated the NTSPNMT neurons innervated by CeA neurons. Scale bar, 20 µm.
