# Supplementary figures and images for: Mapping of afferent and efferent connections of phenylethanolamine N‐methyltransferase‐expressing neurons in the nucleus tractus solitarii
Source: CNS Neurosci Ther. 2024 Jun 17;30(6):e14808. doi: 10.1111/cns.14808 (PMC11183208; doi:10.1111/cns.14808)

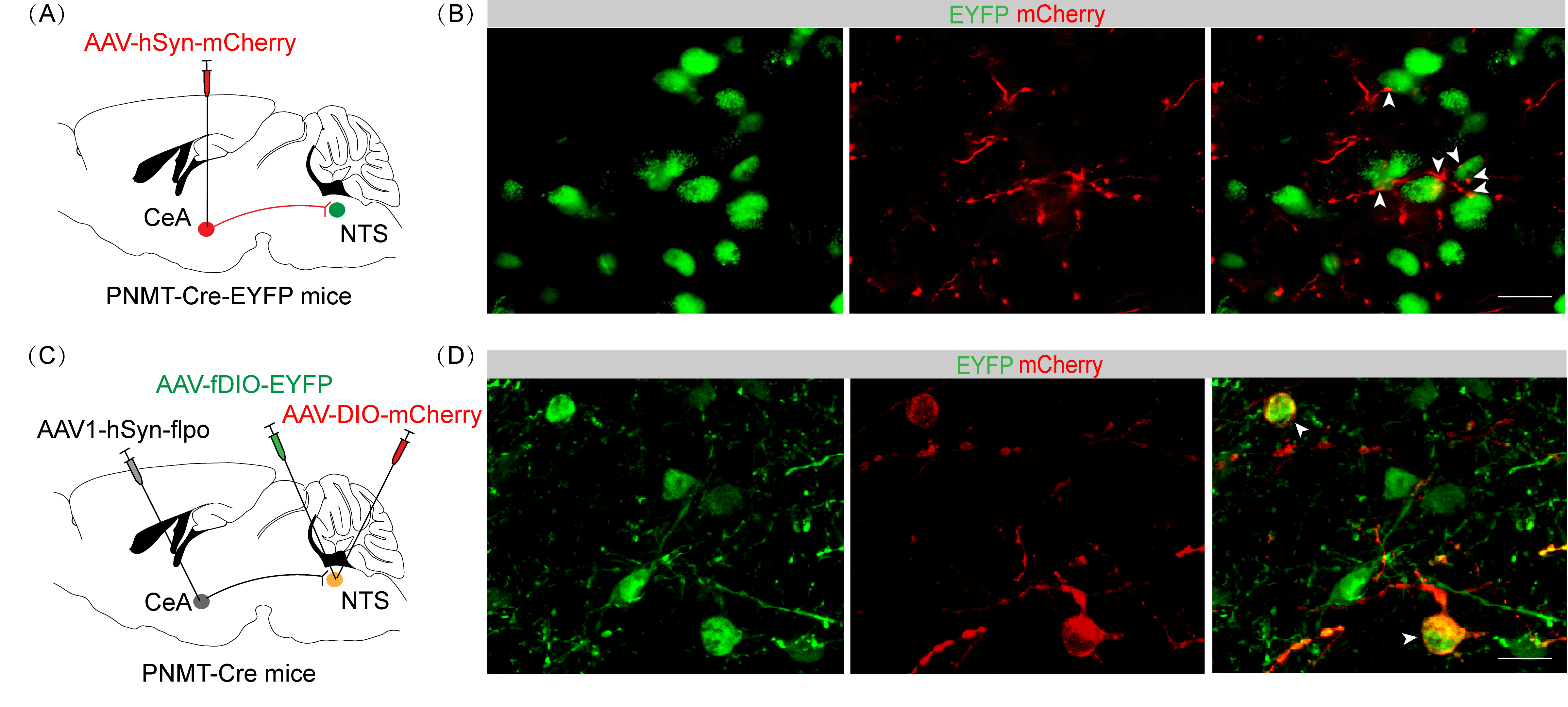

Supplement: Supplementary file 1 — Figure S1. [file CNS-30-e14808-s001.zip › figureS1.tif]
